# Type I collagen facilitates safe and reliable expansion of human dental pulp stem cells in xenogeneic serum-free culture

**DOI:** 10.1186/s13287-020-01776-7

**Published:** 2020-07-14

**Authors:** Mai Mochizuki, Hiroshi Sagara, Taka Nakahara

**Affiliations:** 1 Department of Life Science Dentistry, The Nippon Dental University, 1-9-20 Fujimi, Chiyoda-ku, Tokyo, 102-8159 Japan; 2 Department of Developmental and Regenerative Dentistry, The Nippon Dental University School of Life Dentistry at Tokyo, 1-9-20 Fujimi, Chiyoda-ku, Tokyo, 102-8159 Japan; 3 Medical Proteomics Laboratory, The Institute of Medical Science, The University of Tokyo, 4-6-1 Shirokanedai, Minato-ku, Tokyo, 108-8639 Japan

**Keywords:** Dental pulp, Mesenchymal stem cells, Xenogeneic serum-free culture, Stem cell expansion, Collagen, Cell hypoxia, HIF-1α, Integrin, Mitochondrial dysfunction, Apoptosis

## Abstract

**Background:**

Human dental pulp stem cells (DPSCs) are a readily accessible and promising cell source for regenerative medicine. We recently reported that a xenogeneic serum-free culture medium (XFM) is preferable to fetal bovine serum-containing culture medium for ex vivo expansion of DPSCs; however, we observed that, upon reaching overconfluence, XFM cells developed a multilayered structure and frequently underwent apoptotic death, resulting in reduced cell yield. Therefore, we focused on optimization of the XFM culture system to avoid the undesirable death of DPSCs.

**Methods:**

We selected type I collagen (COL) as the optimal coating substrate for the cultureware and compared DPSCs cultured on COL in XFM (COL-XFM cells) to the conventional XFM cultures (XFM cells).

**Results:**

Our results demonstrated that COL coating facilitated significantly higher rates of cell isolation and growth; upon reaching overconfluence, cell survival and sustained proliferative potential resulted in two-fold yield compared to the XFM cells. Surprisingly, after subculturing the overconfluent COL-XFM cultures, the cells retained stem cell behavior including stable cell growth, multidifferentiation potential, stem cell phenotype, and chromosomal stability, which was achieved through HIF-1α-dependent production and uniform distribution of collagen type I and its interactions with integrins α2β1 and α11β1 at overconfluency. In contrast, cells undergoing apoptotic death within overconfluent XFM cultures had disorganized mitochondria with membrane depolarization.

**Conclusion:**

The use of COL as a coating substrate promises safe and reliable handling of DPSCs in XFM culture, allowing translational stem cell medicine to achieve stable isolation, expansion, and banking of donor-derived stem cells.

## Background

Populations of dental pulp-derived stromal cells contain multipotent stem cells with a high proliferative capacity and potent differentiation potential into a wide range of cell types, such as osteo/odontogenic cells [1–4] and non-dental cells, including neural and endodermal lineages [5–10]. The dental pulp stem cells (DPSCs, also known as dental pulp-derived mesenchymal stem cells [MSCs]) [11, 12], based on their multiple regenerative effects including paracrine signaling, immunomodulation, and immunosuppression [13–16], are considered part of the stem cell compartment and an alternative cell source of medical signaling cells [17, 18]. Dental pulp is a readily accessible donor-tissue resource obtained by routine tooth extraction; therefore, DPSCs present a safe and minimally invasive MSC source for therapeutic applications and cell bank storage for future use [19–22].

It is widely known that the use of xenogeneic serum, i.e., fetal bovine serum (FBS), is necessary for MSC culture to isolate and expand donor-derived stem/stromal cells, including DPSCs [23, 24]. However, serum from an animal source includes potential risks of infection and immunological reaction associated with the cell-based therapies [25, 26]. To avoid FBS, serum-free culture media have been developed and its use has been explored in a variety of MSC types derived from bone marrow [27–29], umbilical cord [30, 31], adipose tissue [32], periodontal ligament [33, 34], and dental pulp [28, 35–38]. It is also recognized that cell isolation from donor-derived tissues (primary culture) is difficult under serum-free conditions. Indeed, previous studies described cell isolation procedures from harvested tissues performed using FBS-containing culture media, and after the primary cultures were established, the subsequent subculture was performed in the serum-free culture media [28–30, 33, 35, 38]. Therefore, it is important to develop an efficient method for isolating MSCs from donor-derived tissues using serum-free culture conditions. Development and application of serum-free MSC culture methodology will ensure safe and reliable cell-based therapies for regenerative medicine [39, 40].

We recently demonstrated a successful isolation and expansion of donor-derived DPSCs under xenogeneic serum-free culture conditions using a commercially available culture medium [37]. Interestingly, the 14-day cell growth curve analysis revealed that DPSCs reached a plateau on day 8 post-seeding and the number of cells declined on day 14. Further analysis revealed that overconfluence of cells on day 14 resulted in increased apoptosis of DPSCs, providing an explanation for the reduced cell yield. Moreover, the overconfluent DPSCs failed to proliferate after passaging. These results suggested that the serum-free cultured cells should be passaged at subconfluence and/or confluence and a prolonged culture to overconfluence should be avoided.

The purpose of this current study was to improve the serum-free culture conditions and to create a reliable method for isolation and expansion of DPSCs. Our previous study revealed that the time required for the initial attachment of primary DPSCs cultured in xenogeneic serum-free culture medium (XFM) was significantly shorter in fibronectin-coated culture dishes compared to the non-coated ones [37]. Therefore, we decided to focus on a widely accepted method of coating culture dishes with common matrix substrates, a safe and simple way to address possible safety concerns for the future clinical applications.

## Methods

### Cell culture

Human dental pulp tissue was obtained from eight healthy volunteers aged 22 to 40 years undergoing routine extraction of third molars and premolars for orthodontic reasons. The isolation and expansion of DPSCs were performed as previously described [24, 37]. Briefly, the enzymatically isolated cells were cultured in XFM (PRIME-XV MSC Expansion XSFM; Irvine Scientific, Santa Ana, CA, USA) in a humidified incubator at 37 °C and 4.7% CO_2_. Upon reaching 80% confluence, the cells were dissociated using 0.25% trypsin–0.02% ethylenediaminetetraacetic acid (EDTA) buffer and then subcultured at a cell density of 5 × 10^3^ cells/cm^2^ in 60-mm culture dishes coated with the substrates as determined by the following pilot experiment. All experiments were performed with cell passages P3–P5. To compensate for donor variability, all experiments were independently repeated using cells obtained from at least four individuals, with the exception of the transmission electron microscope (TEM) analysis.

### Initial cell growth test for determining optimal coating substrate

In the pilot experiment, the culture dishes were coated with the following substrates: type I collagen (Cellmatrix I; Nitta Gelatin, Osaka, Japan and Corning BioCoat; Corning, NY, USA), fibronectin (PRIME-XV Human Fibronectin; Irvine Scientific and Corning BioCoat; Corning), laminin (Corning BioCoat; Corning and Bio-coat; BD Biosciences, San Jose, CA, USA), and poly-d-lysine (Corning BioCoat; Corning). For the initial cell growth test, the cells were seeded at 5 × 10^3^ cells/cm^2^ into respective substrate-coated culture dishes and then observed on day 4 post-seeding (D4) under the microscope. Based on the results of initial cell growth test, the culture dishes coated with the selected substrate were used in subsequent experiments. For cell adherence test, cells were seeded at 2 × 10^3^ cells/cm^2^ and then counted in five randomly selected fields on day 2 post-seeding (D2) using a Biorevo BZ-9000 microscope (Keyence, Osaka, Japan).

### Cell growth curve

The cell growth curve was evaluated as previously described [37]. Briefly, cells were seeded at 5 × 10^3^ cells/cm^2^ into 12-well plates and cultured for 20 days. To generate a cell growth curve, the cells were counted in triplicate using a hemocytometer every 2 days and the results were presented as the mean ± standard deviation (SD).

### Histological examination and immunohistostaining

Cells were cultured in 60-mm dishes for 10, 14, and 20 days post-seeding (D10, D14, and D20), the time points corresponding to confluence (D10) or overconfluence (D14 and D20), and then fixed with 4% paraformaldehyde (PFA) for 10 min at room temperature (RT). Using fine tweezers, the fixed cultures were carefully removed from the dishes, embedded in paraffin, sectioned serially at 5-μm thickness, and stained with hematoxylin and eosin (HE) as previously described [37]. Immunohistostaining was performed according to the published protocol [41] with minor modifications. Briefly, the paraffin sections were prepared as described earlier. For antigen retrieval, sections were deparaffinized, incubated at 95 °C for 20 min in DAKO Target Retrieval Solution (Agilent Technologies, Santa Clara, CA, USA), and then blocked with Blocking One Histo (Nacalai Tesque, Kyoto, Japan) for 10 min at RT. The primary antibodies used were as follows: hypoxia-inducible factor-1 alpha (HIF-1α) (1:100; BD Biosciences), collagen type I alpha 1 (1:1000; Cell Signaling Technology, Danvers, MA, USA), collagen type IV (1:500; Abcam, Cambridge, UK), collagen type VII (1:500; Abcam), and fibronectin (1:200; Proteintech, Rosemont, USA). The secondary antibodies used were as follows: Alexa 488-conjugated donkey anti-mouse immunoglobulin (Ig) G antibody and Alexa 568-conjugated goat anti-rabbit IgG antibody (both diluted 1:1000; Life Technologies, Carlsbad, CA, USA). The stained sections were mounted with Fluoro-KEEPER Antifade Reagent containing 4′,6-diamidino-2-phenylindole (DAPI) (Nacalai Tesque) and examined using a confocal laser-scanning microscope (LSM 700; Carl Zeiss, Oberkochen, Germany). Sections incubated without primary antibodies were used as negative controls.

### Stem cell characterization

To characterize subcultured cells for stem cell properties (stemness) after passaging of the overconfluent cultures, we assessed stem cell marker expression, multidifferentiation potential, and chromosomal stability. For marker expression analyses, the cell surface antigens and mRNA expression were evaluated by flow cytometry and reverse transcription-polymerase chain reaction (RT-PCR), respectively. For flow cytometric analysis, the following antibodies were used: CD14-FITC (clone M5E2), CD29-APC (clone MAR4), CD73-FITC (clone AD2), CD90-APC (clone 5E10), CD105-PE (clone 266) (all from BD Biosciences), CD34-FITC (clone 581), and CD44-FITC (clone J.173) (both from Beckman Coulter, Fullerton, CA, USA). For RT-PCR, total RNA was extracted from cell cultures and analyzed as previously described [37]. Primer sequences and PCR conditions are summarized in Table 1. Multidifferentiation induction culture into osteogenic and adipogenic lineages was evaluated as previously described [42].

**Table 1:** Primer sequences and amplification conditions for RT-PCR analysis

Gene	Primer sequences, 5′ to 3′		Product size (bp)	Annealing temperature (°C)	GenBank accession number
*Vimentin*	S:	GGGACCTCTACGAGGAGGAG	200	55	NM_003380
	A:	CGCATTGTCAACATCCTGTC			
*Nanog*	S:	ACCTTCCAATGTGGAGCAAC	199	55	NM_024865
	A:	GAATTTGGCTGGAACTGCAT			
*Oct3/4*	S:	GACAGGGGGAGGGGAGGAGCTAGG	144	60	NM_001173531
	A:	CTTCCCTCCAACCAGTTGCCCCAAAC			
*Sox2*	S:	AACCCCAAGATGCACAACTC	152	60	NM_003106
	A:	CGGGGCCGGTATTTATAATC			
*Runx2*	S:	CCCCACGACAACCGCACCAT	292	55	NM_001024630
	A:	GTCCACTCCGGCCCACAAATC			
*COL1*	S:	CCAAATCTGTCTCCCCAGAA	214	55	NM_000088
	A:	TCAAAAACGAAGGGGAGATG			
*Nestin*	S:	AACAGCGACGGAGGTCTCTA	220	55	NM_006617
	A:	TTCTCTTGTCCCGCAGACTT			
*GAPDH*	S:	GTCAAGGCTGAGAACGGGAA	613	55	NM_001289746
	A:	GCTTCACCACCTTCTTGATG			
*β-actin*	S:	GGACTTCGAGCAAGAGATGG	234	60	NM_001101
	A:	AGCACTGTGTTGGCGTACAG			

*Oct3/4* POU class 5 homeobox 1 (POU5F1), *SOX2* sex-determining region Y-box 2, *Runx2* runt-related transcription factor 2, *COL1* collagen type I, *GAPDH* glyceraldehyde-3-phosphate dehydrogenase

### Karyotype analysis

Cells grown to the mid-logarithmic stage were subjected to standard G-banding chromosome analysis at the Nihon Gene Research Laboratories (Sendai, Japan).

### Detection of intracellular hypoxia

To visualize intracellular hypoxia in overconfluent cultures on D20, we used the hypoxia probe LOX-1 (SCIVAX, Kanagawa, Japan), which has its phosphorescence quenched by oxygen, as previously described [43]. Briefly, the cultures were incubated in the presence of 2 μM LOX-1 for 1 h in a humidified incubator and then visualized by a fluorescent microscope (Keyence).

### Single-cell analysis of HIF-1α localization in cobalt chloride (CoCl_2_)-induced hypoxia

To validate HIF-1α nuclear localization, cells were incubated with 100 μM CoCl_2_ for 24 h to induce hypoxia. Immunocytostaining was performed as previously described [41]. The following primary antibodies were used: HIF-1α (1:500; GeneTex, Irvine, CA, USA) and vimentin (1:10,000, Merck KGaA, Darmstadt, Germany). The following secondary antibodies were used: Alexa 568-conjugated goat anti-mouse IgG antibody and Alexa 488-conjugated goat anti-rabbit IgG antibody (both diluted 1:1000; Life Technologies). All images were acquired using the same settings on the confocal microscope (Carl Zeiss) and analyzed using ZEN 3.0 software (blue edition; Carl Zeiss). Samples incubated without a primary antibody were used as a negative control.

### Cell cycle analysis by flow cytometry

The cell cycle analysis was performed using flow cytometry as previously described [24]. Flow cytometry was performed using BD FACSMelody cell sorter equipped with BD FACSChorus software (BD Biosciences). The percentage of the cells in the G0/G1, S, or G2/M phase was measured using FlowJo v10 software (Tree Star, Ashland, OR, USA).

### Western blotting

Western blotting was performed as previously described [44] with minor modifications. Briefly, cell cultures were lysed in cell lysis buffer (10 mM Tris-HCl (pH 7.4), 1 mM MgCl_2_, 0.1% Triton X-100) and the total amount of soluble proteins was quantified using the BSA protein assay kit (Thermo Fisher Scientific, Waltham, MA, USA). The cell lysates were separated by sodium dodecyl sulfate-polyacrylamide gel electrophoresis and transferred to a polyvinylidene fluoride membrane. Membranes were blocked with Blocking One (Nacalai Tesque) for 30 min at RT. The primary antibodies were collagen type I alpha 1 and β-actin (both diluted 1:1000; Cell Signaling Technology), and Ki67 (1:2000; Abcam). The secondary antibodies were horseradish peroxidase (HRP)-conjugated goat anti-rabbit IgG antibody and HRP-conjugated horse anti-mouse IgG antibody (both diluted 1:2000; Cell Signaling Technology). The chemiluminescence was detected using AE-9300 Ez-Capture MG imaging system (ATTO, Tokyo, Japan). Density of protein bands was quantified by ImageJ software (ver. 1.52a; National Institutes of Health, Bethesda, MD, USA) and the results were normalized to β-actin. The values are presented as mean ± SD (*n* = 4).

### HIF-1α inhibition by YC-1 in CoCl_2_-induced hypoxia

To validate the effect of YC-1, a HIF-1α inhibitor (Santa Cruz Biotechnology, Santa Cruz, CA, USA), cells were cultured with 100 μM YC-1 for 24 h in CoCl_2_-induced hypoxic conditions and HIF-1α nuclear localization was assessed. Next, to investigate whether HIF-1α regulated collagen type I (COL1) gene expression in our system, we treated cells cultured in CoCl_2_-induced hypoxic conditions (12-well plates, 3 × 10^5^ cells/cm^2^) with 100 μM YC-1 for 24 h. COL1 gene expression was assessed by RT-PCR.

### TEM analysis

The overconfluent cell cultures were fixed in 100 mM phosphate buffer solution (PB) containing 2% PFA and 1% glutaraldehyde for 2 h at RT. Sections of the cell layers were dissected out, postfixed in PB containing 2% osmium tetroxide, dehydrated in graded series of ethanol, and embedded in Epon 812 (TAAB, England, UK) resin mixture. Ultrathin sections were cut by an ultramicrotome, stained with uranyl acetate and lead citrate. The sections were examined using transmission electron microscope (Hitachi H-7500, Tokyo, Japan).

### Mitochondrial membrane potential

The mitochondrial membrane potential (ΔΨm) was assessed by flow cytometry using the JC-1 MitoScreen assay (BD Biosciences) according to the manufacturer’s instructions. Flow cytometry on JC-1-stained cells was performed using BD FACSMelody cell sorter equipped with BD FACSChorus software (BD Biosciences). Single-cell samples were gated to exclude cell debris and aggregates using FlowJo v10 software (Tree Star). The quantification of the JC-1 monomers fold change from D10 to D20 in each cell type was determined using the following formula: the ratio of JC-1 monomers to JC-1 aggregates on D20 was divided by the corresponding ratio on D10. The values were expressed as mean ± SD (*n* = 4).

### Terminal deoxynucleotidyl transferase deoxyuridine triphosphate nick end labeling (TUNEL) staining

The deparaffinized sections of cell cultures fixed on D10, D14, and D20 were prepared as described earlier. TUNEL staining was performed according to the manufacturer’s instructions (Roche Diagnostics, Mannheim, Germany). The stained sections were examined using a confocal microscope (Carl Zeiss).

### Statistical analysis

Statistical analyses were performed using IBM SPSS Statistics software (version 23.0; IBM Japan, Tokyo, Japan). For a two-group comparison, an unpaired *t* test was applied. Mann–Whitney *U* test was used for nonparametric data. For all statistical analyses, *p* < 0.05 indicated statistical significance.

## Results

### Type I collagen facilitates initial cell growth under XFM culture conditions

To investigate the benefits of coating cultureware to improve XFM culture conditions, first, we tested several commonly used substrates, including type I collagen (COL), fibronectin, laminin, and poly-d-lysine. After a 4-day incubation period, DPSCs grew in culture plates coated with COL and fibronectin, while the cells did not adhere in the plates coated with laminin and poly-d-lysine (Additional file 1: Figure S1). In the present study, DPSCs cultured on COL in XFM were designated as “COL-XFM cells”, while DPSCs cultured on fibronectin in XFM were designated as conventional “XFM cells” [37].

To further characterize the substrates, the cells isolated from the dental pulp tissue by enzymatic digestion were seeded on COL or fibronectin and the adherent cells were evaluated as previously described [37]. We observed that the time period required to show cell division of adherent cells in COL-XFM primary culture was significantly shorter compared to the XFM primary culture (4.0 ± 1.0 vs 7.8 ± 1.3 days; *n* = 4; *P* = 0.0073). Furthermore, after seeding cells on COL at low cell density, COL-XFM cells had a significantly higher number of adhered cells compared to the XFM cells after 2 days of culture (*p* < 0.01; Additional file 2: Figure S2). In summary, COL provided consistent cell isolation and growth at the initial cell proliferation phase in XFM culture conditions. Therefore, we selected COL as the optimal coating substrate for our subsequent experiments.

### COL facilitates robust cell growth and prolonged cell expansion

**Fig. 1:**
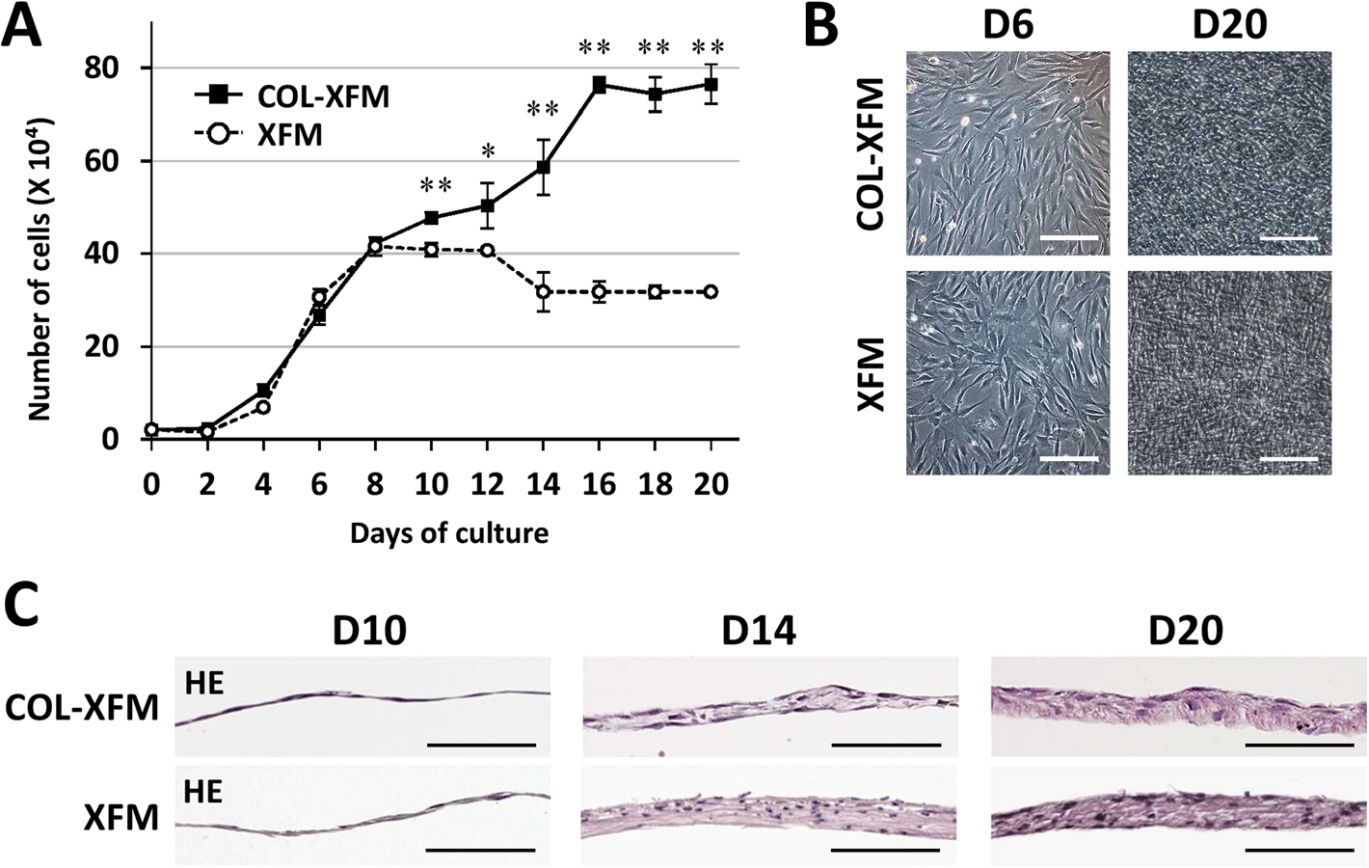
Cell growth dynamics of DPSCs cultured on type I collagen (COL) under xenogeneic serum-free medium (XFM) culture conditions. **a** Growth curve of COL-XFM and XFM cells during 20 days of culture. *p < 0.05; **p < 0.01. **b** Phase-contrast images of COL-XFM and XFM cells on day 6 (D6; the logarithmic phase) and on day 20 (D20; the overconfluent phase) post-seeding. **c** Histology of COL-XFM and XFM cultures on day 10 (D10; confluence) and on days 14 and 20 (D14 and D20; overconfluence); hematoxylin and eosin staining. All scale bars, 100 μm

To characterize the growth curves of COL-XFM and XFM cells, we cultured the cells for up to D20. XFM cells showed active growth and reached a plateau during 12 days of culture; the number of cells decreased on D14 as previously reported [37] and remained constant until D20 (Fig. 1a). Interestingly, COL-XFM cells exhibited robust and sustained growth and reached a plateau on day 16 without any reduction of cell number throughout 20 days of culture, yielding double the number of cells compared to the XFM cells (Fig. 1a). Phase-contrast observation on D6 and D20 and the histological examination on D10 showed no obvious differences in both cultures (Fig. 1b, c). Both cell types developed a multilayered structure by D14 and produced abundant extracellular matrix (ECM) by D20, as demonstrated by the eosin staining (Fig. 1c). Furthermore, we observed the condensed nuclei in the XFM cells on D14 and D20, but not in the COL-XFM cells. At the same time, the COL-XFM multilayered structure became thicker between D14 and D20, while the XFM tissue thickness did not appear to change during that time period (Fig. 1c). In summary, we found that under XFM culture conditions, COL facilitates robust cell growth of DPSCs even after overconfluence and the development of well-organized multilayered ECM.

### The multilayered COL-XFM cells maintain stable growth and stem cell properties after subculture

**Fig. 2:**
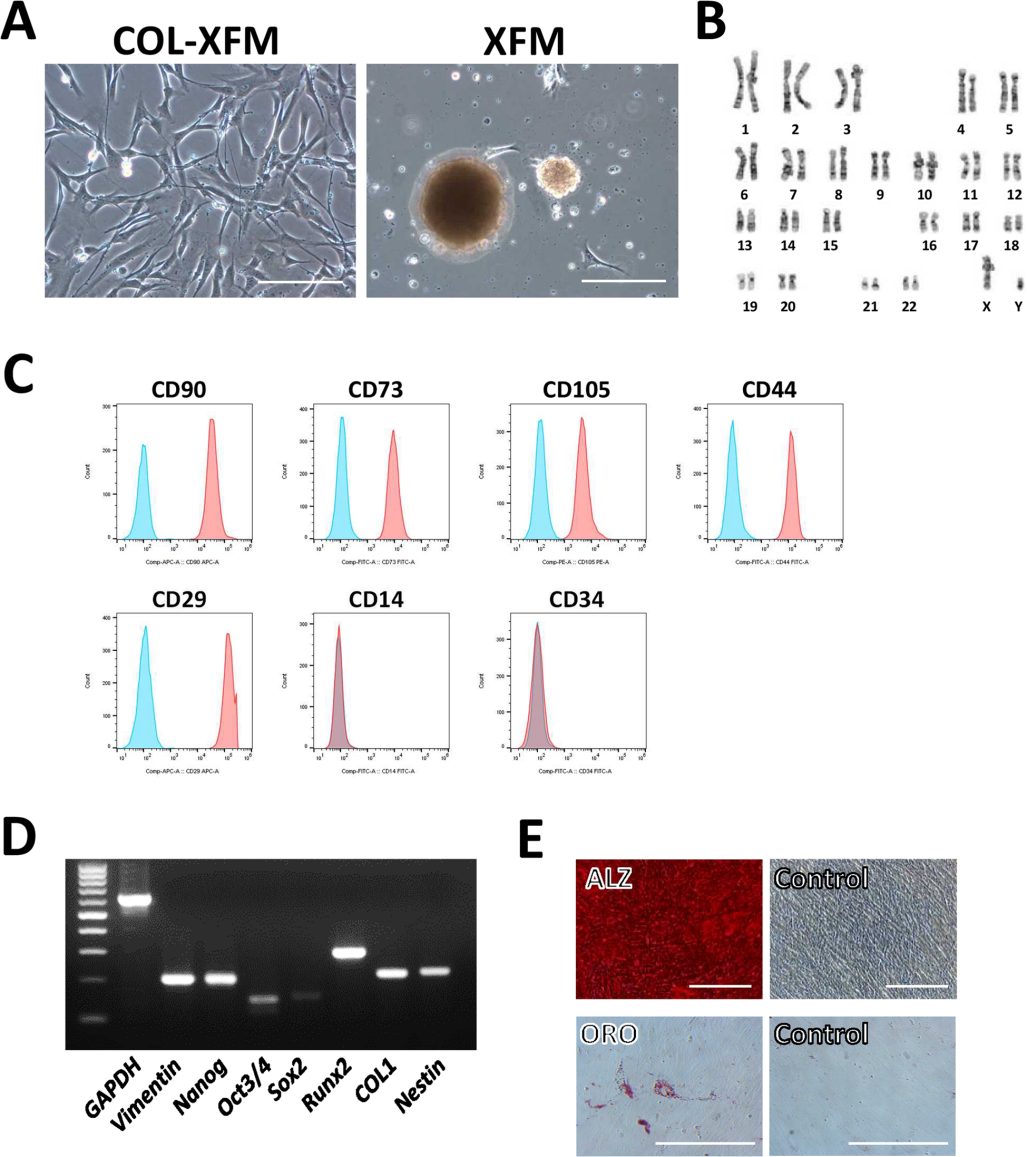
Stem cell characterization after subculture of overconfluent/multilayered COL-XFM cells. **a** Phase-contrast images of the subcultured COL-XFM and XFM cells. Scale bars, 100 μm. The subcultured COL-XFM cells were subjected to the following evaluations: **b** karyotyping, **c** evaluation of common stem cell marker antigens by flow cytometry, **d** reverse transcription-polymerase chain reaction (RT-PCR) to confirm gene expression profile of common stem cell markers, and **e** confirmation of multidifferentiation potential: cells were differentiated into osteogenic and adipogenic lineages as determined by alizarin red staining (ALZ) and oil red O staining (ORO), respectively. Scale bars, 100 μm in ALZ and control images in upper panels; 50 μm in ORO and control images in lower panels

In our previous study, we reported that, upon passaging the overconfluent XFM cultures on D14, XFM cells formed aggregates consisting of senescent or dead cells, and lost proliferative capacity [37]. To investigate the effect of COL coating on cell growth and stem cell properties, both XFM multilayers were subcultured on D20. Surprisingly, COL-XFM cells were passaged normally and showed stable subsequent proliferation (Fig. 2a). In contrast, XFM cells formed aggregates and did not proliferate any further (Fig. 2a), consistent with our previous study [37]. Furthermore, the subcultured COL-XFM cells showed normal karyotype with diploidy (Fig. 2b), presence of common stem cell marker antigens (Fig. 2c), typical gene expression profile (Fig. 2d), and multidifferentiation potential (Fig. 2e), all of the parameters consistent with the DPSC properties [24, 37]. In summary, these results indicated that after subculture, the multilayered COL-XFM cells maintained stem cell properties (stemness), including the proliferative ability, multidifferentiation potential, and stem cell phenotype along with chromosomal stability.

### COL coating induces nuclear localization of HIF-1α and proliferative potential in COL-XFM cells

**Fig. 3:**
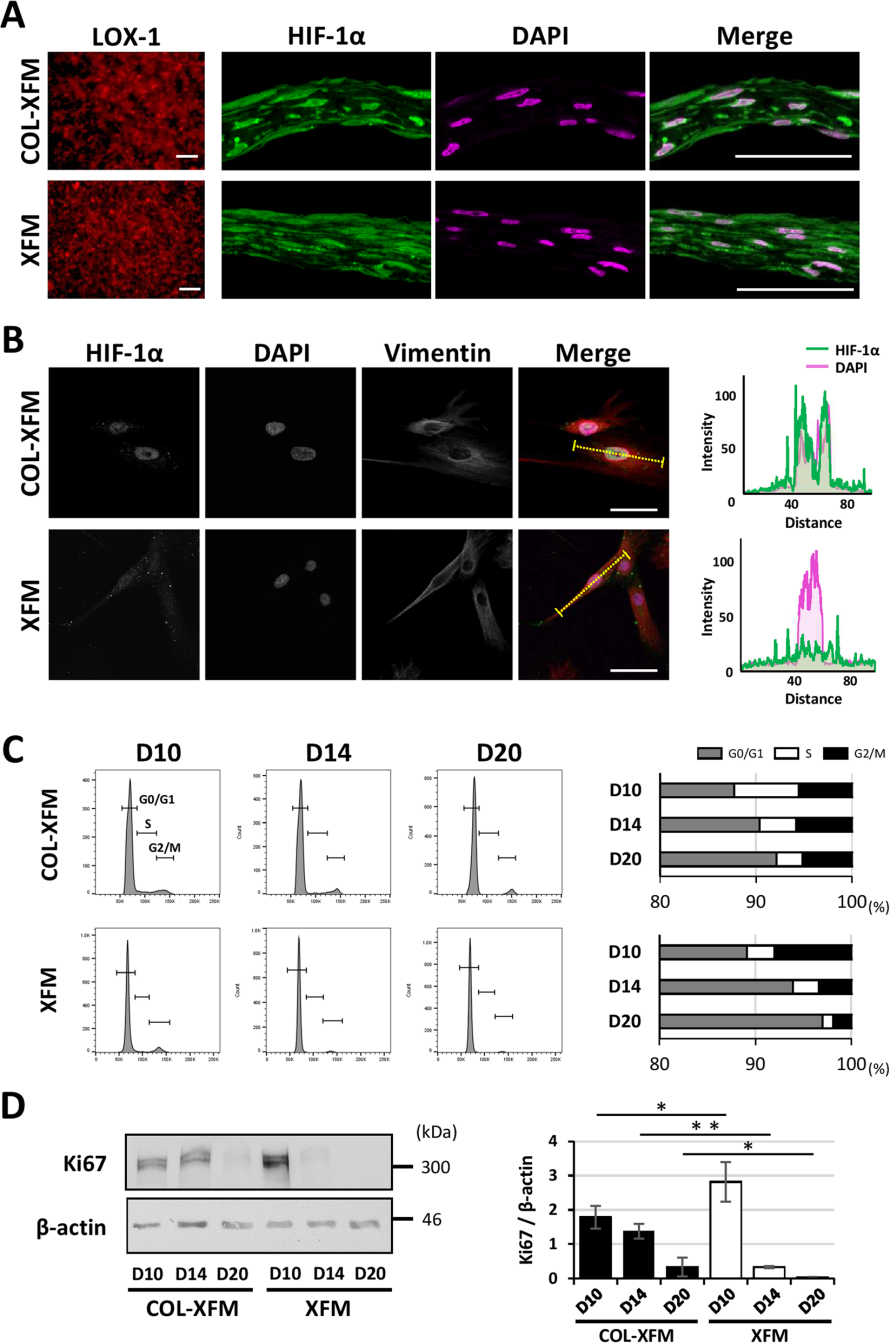
Hypoxia probe (LOX-1), HIF-1α expression, localization, cell cycle, and Ki67-expression analyses of COL-XFM and XFM cultures. **a** LOX-1 phosphorescence and HIF-1α immunohistostaining of the multilayered COL-XFM and XFM cultures on D20. All nuclei are shown in magenta (DAPI). Scale bars, 100 μm in the panels of LOX-1 and 50 μm in the panels labeled “Merge.” **b** Immunocytostaining of COL-XFM and XFM cells treated with cobalt chloride to induce hypoxia; single-cell analysis of HIF-1α localization. The single channels (HIF-1α, DAPI, and vimentin) are shown in gray. The fluorescence intensities of HIF-1α (green) and nuclei (DAPI, magenta) are indicated by the dashed line (yellow) depicted in a single cell; cell morphology was visualized using vimentin staining (red on merged images). Scale bars, 50 μm. **c** Cell cycle analysis using flow cytometry in COL-XFM and XFM cells on days 10 (D10; confluence), 14, and 20 (D14 and D20; overconfluence) post-seeding. Representative histograms are shown. The percentage of the cells in G0/G1, S, and G2/M phases of cell cycle is quantified and presented in the panel on the right. **d** Western blot and quantification of Ki67 expression in COL-XFM and XFM cells on D10, D14, and D20. β-actin was used as an internal control to normalize protein expression of Ki67. *p < 0.05; **p < 0.01

To investigate the mechanisms regulating cells at overconfluence, first, we assessed the hypoxic conditions in D20 multilayered cultures using LOX-1 staining. As expected, LOX-1 phosphorescence, an indication of a hypoxic state, was detected in both XFM and COL-XFM multilayers (Fig. 3a). Next, we hypothesized that HIF-1α, a master hypoxia regulator, plays an important role in the cellular responses in COL-XFM and XFM multilayers. Immunohistostaining revealed that HIF-1α was localized predominantly in the nuclei of the COL-XFM multilayer, whereas in the XFM multilayer, HIF-1α was also detected in the cytoplasm (Fig. 3a). To confirm nuclear localization of HIF-1α, we performed a single-cell analysis of COL-XFM and XFM cells. Cells were seeded at a low cell density and treated with CoCl_2_ to mimic the hypoxic conditions. As expected, HIF-1α was localized to the nucleus of the COL-XFM cell, and the fluorescence intensity of HIF-1α overlapped with DAPI staining (Fig. 3b). By contrast, in the XFM cell, HIF-1α was also detected in the cytoplasm and not exclusively in the nucleus (Fig. 3b). In summary, our results demonstrated that nuclear localization of HIF-1α can be induced by hypoxic conditions in COL-XFM cultures, suggesting that HIF-1α could be involved in cellular responses at overconfluency.

It is well known that HIF-1α is a potent proliferative regulator under hypoxia [45, 46]. Therefore, we decided to evaluate the cell cycle status and time-course expression of Ki67, a proliferation marker tightly linked to the cell cycle, in COL-XFM and XFM cells on D10 (confluence), D14, and D20 (overconfluence). First, we performed a cell cycle analysis using flow cytometry. A considerable percentage of COL-XFM cells was in the G2/M and S phases on D10, D14, and D20. In contrast, the percentage of XFM cells in the G2/M and S phases was markedly decreased on D14 and thereafter, resulting in the cell cycle arrest in the G0/G1 phase (Fig. 3c). This was further verified by western blotting. COL-XFM cells showed stable expression of Ki67 on D10 and D14, with low expression levels still detectable on D20. At the same time, in the XFM cells, the Ki67 expression was observed on D10, but it was dramatically downregulated on D14 and was almost undetectable on D20 (Fig. 3d). These results suggested that the robust cell growth after reaching confluence in COL-XFM cells was regulated by active cell cycle status and sustained expression of Ki67; however, the cell cycle arrest and lack of Ki67 expression resulted in decreased growth of the XFM cells on D14 and thereafter.

### Integrins α2β1- and α11β1-mediated interactions with COL1, regulated by HIF-1α, are responsible for cell survival in COL-XFM cells during prolonged culture

**Fig. 4:**
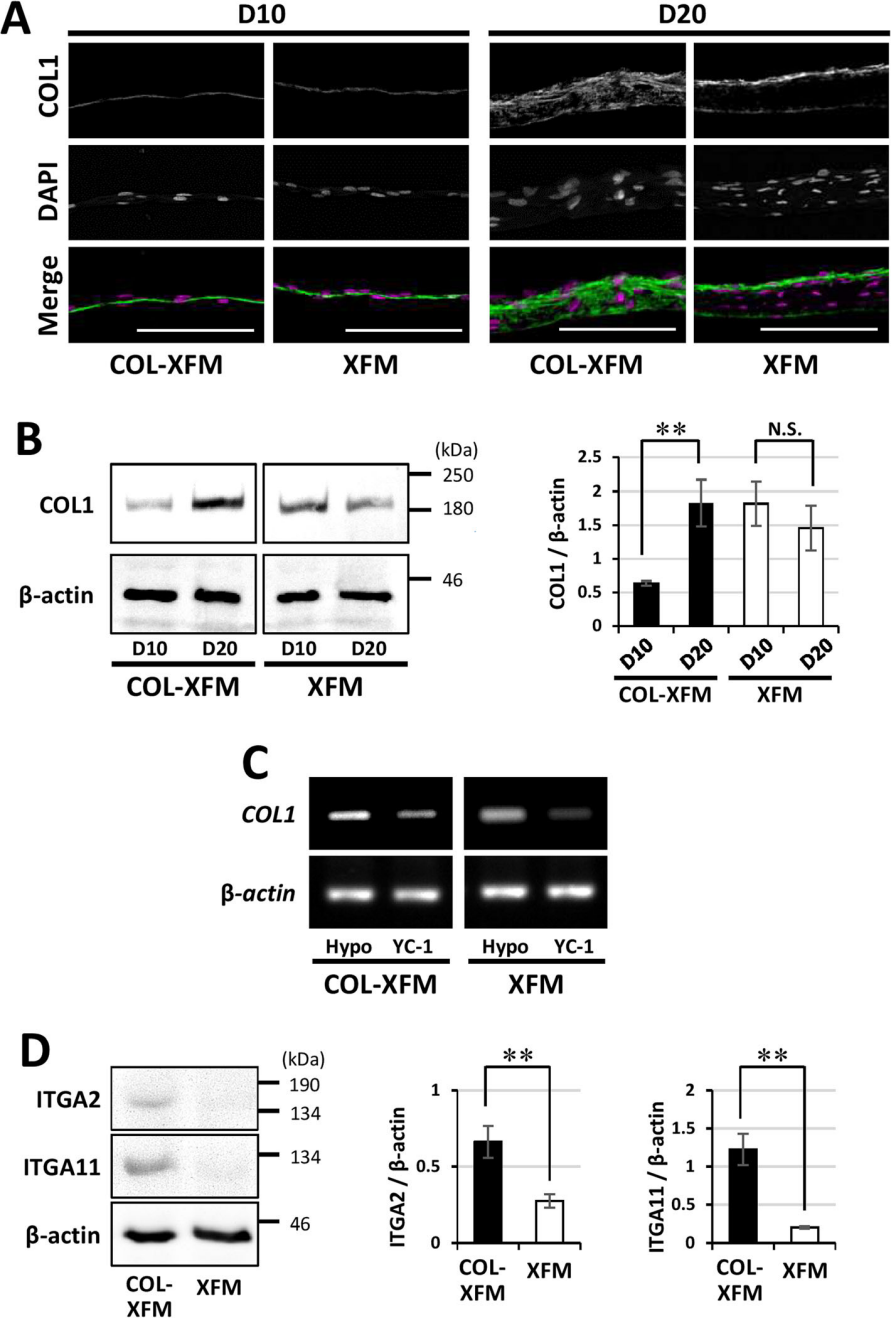
Collagen type I (COL1) expression, regulation, and interactions in COL-XFM and XFM cultures on D10 and D20. **a** Immunohistostaining of both confluent/monolayered and overconfluent/multilayered XFM cultures on D10 and D20, respectively. The single channels (COL1 and DAPI) are shown in gray; all nuclei in the merged images are shown in magenta (DAPI); scale bars, 100 μm. **b** Immunoblotting and quantification of COL1 protein expression in the cultures described in **a**. β-actin was used as a loading control to normalize protein expression of COL1; **p < 0.01; N.S., no significant difference. **c** RT-PCR was used to measure gene expression of *COL1* in COL-XFM and XFM cells in the absence (Hypo) or presence (YC-1) of a HIF-1α inhibitor YC-1 during cobalt chloride-induced hypoxia. **d** Western blot and quantification of integrin α2 and α11 subunits (ITGA2 and ITGA11) in both types of multilayered XFM cultures on D20. β-actin was used as a loading control to normalize the expression of ITGA2 and ITGA11. **p < 0.01

To investigate whether the ECM composition was different between the two XFM cultures, we focused on COL1, the major component of ECM. Immunohistostaining revealed that on D10 there was no apparent difference in COL1 staining between the two XFM confluent monolayers (Fig. 4a). However, by D20, COL1 staining was stronger and more uniform throughout the COL-XFM multilayer, while the immunoreactivity in the XFM multilayer was limited to the apical (upper) and basal (attached) surfaces and, surprisingly, was completely absent in the intermediate layer (Fig. 4a). Next, we assessed COL1 expression by immunoblotting (Fig. 4b). Our results demonstrated that COL1 levels were significantly increased in COL-XFM cells on D20 compared to D10 (*p* < 0.01), while in the XFM cells, there was no significant difference in COL1 protein levels between D10 and D20.

Next, to elucidate the role of HIF-1α in COL1 production in the XFM cells, we investigated *COL1* mRNA expression in the presence of commonly used HIF-1α inhibitor YC-1 by RT-PCR. First, we tested the effect of YC-1 on HIF-1α nuclear translocation in cells cultured under CoCl_2_-induced hypoxia conditions. As expected, YC-1 completely blocked the nuclear localization of HIF-1α in a COL-XFM cell (Additional file 3: Figure S3). Furthermore, in the presence of YC-1, the expression of *COL1* was markedly decreased in both types of XFM cells (Fig. 4c), suggesting that the production of COL1 was regulated by HIF-1α. We also assessed D20 expression of collagen types IV, VII, and fibronectin, the components of ECM involved in its function and stability. Interestingly, there were no apparent differences in the distribution patterns of these ECM proteins in both multilayers (Additional file 4: Figure S4).

Considering the uneven distribution of COL1 in the XFM multilayer, we hypothesized that the lack of interactions between the cells and the ECM had resulted in the apoptotic death of the cells, a phenomenon known as anoikis. It was reported that integrins α2β1 and α11β1, the specific receptors to COL1, were required for cell death/survival in bone marrow-derived stromal cells (BMSCs) [47]. Therefore, we investigated whether the expression of α2 and α11 integrin subunits was affected in both XFM multilayers. Western blot analysis clearly revealed that the expression of α2 and α11 integrin subunits on D20 was significantly lower in the XFM cells compared to the COL-XFM cells (Fig. 4d). These results suggest that COL1 binding interactions mediated by integrins α2β1 and α11β1 might be responsible for cell survival in COL-XFM multilayer.

### Disorganized mitochondria with membrane depolarization contributed to cell death in overconfluent XFM but not in COL-XFM cultures

**Fig. 5:**
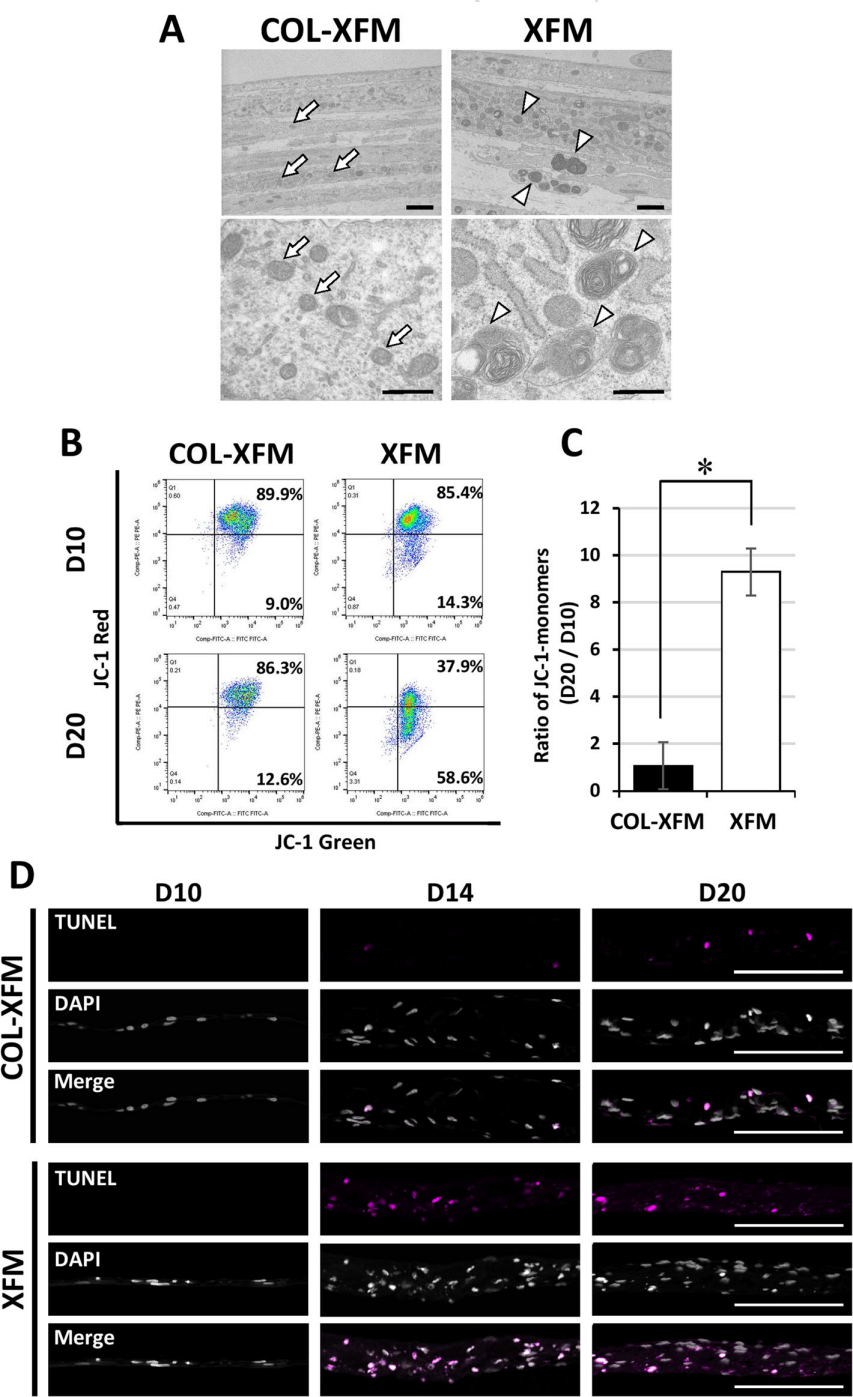
Ultrastructure, cell morphology, and mitochondrial function of DPSCs cultured on COL coating under the XFM culture conditions. **a** TEM of both types of multilayered XFM cultures on D20 post-seeding. Arrows indicate healthy mitochondria; arrowheads indicate disorganized mitochondria. Scale bars, 1.7 μm for the upper panels and 500 nm for the lower panels. **b** JC-1 stained cells were analyzed by flow cytometry to evaluate mitochondrial membrane potential in COL-XFM and XFM cultures on days 10 (D10) and 20 (D20). **c** Quantification of the JC-1 monomers ratio, presented as fold difference of D20 vs. D10 in each cell culture condition. *p < 0.05. **d** Terminal deoxynucleotidyl transferase-mediated deoxyuridine triphosphate nick end labeling (TUNEL) staining of COL-XFM and XFM cultures on D10, D14, and D20. TUNEL-positive nuclei are shown in magenta. All nuclei are shown in gray (DAPI). Scale bars, 100 μm

To explore the cause of cell death in the XFM cultures, we investigated cellular ultrastructure of multilayers on D20 by TEM. As shown in Fig. 5a, both cultures exhibited proper stratified appearance consisting of squamous cells. COL-XFM cells showed normal structure of organelles including the mitochondria, whereas the cytoplasm of the XFM cells had a considerable number of dense structures, containing disorganized mitochondria of different shapes and sizes. These results suggested that the cell death in the XFM multilayer could be caused by the mitochondrial abnormality.

Next, we decided to further investigate cell morphology and mitochondrial function. Both cultures were trypsinized on D20 and cell suspensions were analyzed by flow cytometry to determine the distribution profiles of cell size and internal complexity/granularity based on the forward scatter (FSC-A) and side scatter (SSC-A) dot plots, respectively. The results indicated that COL-XFM cells showed a normal distribution of population with a high homogeneity in both the cell size (FSC-A) and internal granularity (SSC-A) (Additional file 5: Figure S5). In contrast, the majority of the XFM cells were smaller compared to the COL-XFM cells and showed a higher degree of heterogeneity based on the FSC-A dot plot. Furthermore, based on the SSC-A profile data, XFM cells had a heterogeneous distribution of cell internal structures (Additional file 5: Figure S5), possibly due to high cytoplasmic granularity as a result of numerous disorganized mitochondria detected by the TEM (Fig. 5a).

To assess mitochondrial health, we used JC-1 fluorescent dye to analyze mitochondrial membrane potential by flow cytometry. First, the appropriate gates were set to include cells while excluding cell debris and aggregates (Additional file 6: Figure S6A). COL-XFM cells cultured for 1 week without media change were used as a positive control to verify depolarized (unhealthy) mitochondria, while unstained cells served as a negative control (Additional file 6: Figure S6B). Our results demonstrated that there were no differences in distribution of JC-1 aggregates in COL-XFM cells at both D10 (confluence) and D20 (overconfluence) time points, whereas the distribution of JC-1 aggregates dramatically decreased in XFM cells between D10 and D20 (Fig. 5b). We quantified the ratio of the JC-1 monomers on D20 to those on D10 and demonstrated that the ratio was significantly higher in XFM cells compared to the COL-XFM samples (*p* < 0.05; Fig. 5c). These results indicated that mitochondrial depolarization in XFM cells was significantly increased between D10 (confluence) and D20 (overconfluence), while COL-XFM cells maintained healthy mitochondria with stable membrane potential even after confluence. Based on these results, we decided to examine the level of apoptosis in both cultures. As expected, multilayered XFM cells on D14 and D20 appeared to have a higher number of apoptotic cells, as evaluated by TUNEL staining, compared to the COL-XFM multilayer (Fig. 5d). In summary, disorganized mitochondria with membrane depolarization contributed to cell death in overconfluent XFM cultures; however, the presence of COL prevented such changes in COL-XFM cells.

## Discussion

Here, in this study, we present evidence that COL-XFM culture promoted initial attachment and subsequent growth without a reduction in cell yield during a prolonged 20-day culture; however, the conventional XFM culture showed growth arrest upon reaching a plateau and a declined cell yield due to apoptotic cell death and mitochondrial dysfunction. Regarding the mechanisms regulating the distinct outcomes, we demonstrated that HIF-1α played an important role during overconfluence, a hypoxic microenvironment, as shown by HIF-1α-nuclear localization in COL-XFM cells. The assay using the HIF-1α inhibitor YC-1 further confirmed that the expression of *COL1* was regulated by HIF-1α. As a result, HIF-1α significantly upregulated COL1 production in the COL-XFM multilayer, but not in the XFM multilayer, as shown by immunostaining and immunoblotting (Fig. 4). At the same time, expression and spatial distribution of other ECM proteins, including collagen types IV, VII, and fibronectin, were not affected in both types of XFM multilayers (Additional file 4: Figure S4). These results suggested that HIF-1α-dependent COL1 production and its distribution within the multilayered COL-XFM structure were essential for cell survival and proliferative potential in the hypoxic environment. Our results also demonstrated that cell fate and function were closely associated with the collagen–integrin interactions, as discussed below.

In general, upon establishing cell adhesion to ECM, the integrins activate several intracellular signaling pathways involved in the regulation of cell proliferation, migration, differentiation, as well as survival and death [48–50]. In particular, cell adhesion to COL1 is mediated by integrins α1β1, α2β1, and α11β1 [51]. Previous study reported that in BMSCs, interference with COL1 binding to the integrins α2β1 and α11β1 resulted in cell death (anoikis) due to mitochondrial depolarization (this study was performed in the presence of FBS) [47, 52]. In line with these findings, we observed that multilayered XFM cultures showed significantly lower expression of α2 and α11 integrin subunits compared to the multilayered COL-XFM cultures (Fig. 4d). Our findings also showed that the lack of α2β1/α11β1-mediated interactions with COL1 resulted in cell death accompanied by disorganized mitochondria with membrane depolarization (Fig. 5). As demonstrated in the overconfluent/multilayered COL-XFM cultures, COL1-rich microenvironment likely provided the stem cell niche within the multilayered structure. Therefore, COL1–integrins α2β1/α11β1 interactions were essential for not only cell survival, but also for stemness maintenance in COL-XFM cells. In contrast, XFM cells were unable to establish appropriate α2β1/α11β1-mediated interactions, because of the lack of COL1, resulting in anoikis. Given that collagen production, cell survival, and self-renewal ability in hypoxia were regulated by HIF-1α [46, 53, 54], overconfluent COL-XFM cells avoided apoptotic death and strengthened the proliferative potential as verified by active cell cycle status and sustained Ki67-expression.

**Fig. 6:**
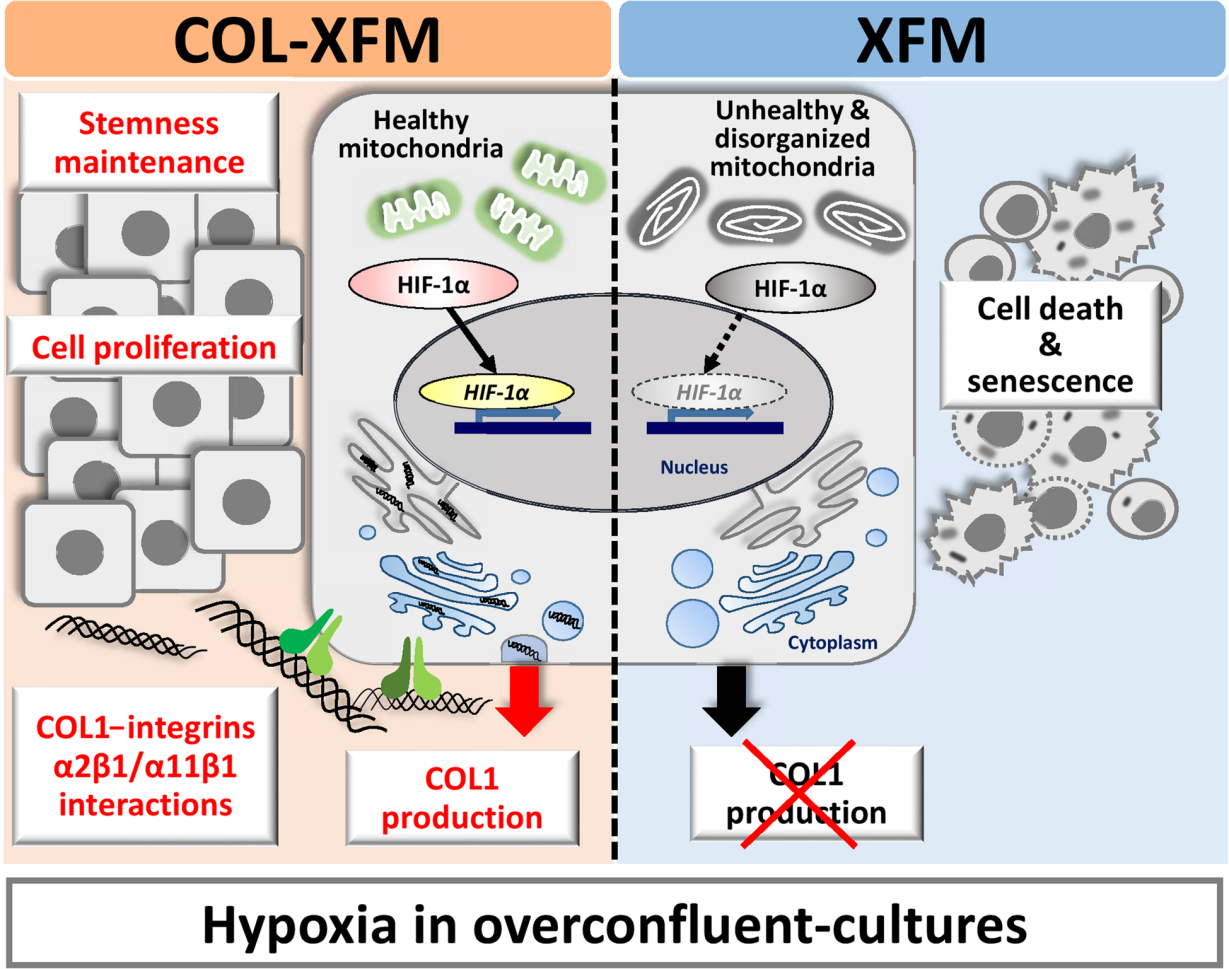
Schematic diagram summarizing the role of ECM, hypoxia, HIF-1α-dependent production of collagen type I (COL1), and integrins α2β1 and α11β1 in cell survival, proliferative potential, and stemness maintenance in DPSCs. In the absence of COL coating, upon reaching overconfluence, the multilayer formation generates the hypoxic environment, leading to apoptotic death accompanied by mitochondrial abnormality in the XFM cells. COL coating promotes cellular attachment, proliferation, and survival of COL-XFM cells. Furthermore, nuclear localization of HIF-1α within the COL-XFM multilayer activates the production of COL1. The resulting COL1-integrins α2β1/α11β1 interactions lead to cell survival and increased proliferative potential. Upon passaging, the COL-XFM cells retain stemness (i.e., proliferative ability, multidifferentiation potential, and stem cell phenotype) and chromosomal stability. Without COL coating, XFM cells display apoptosis and mitochondrial abnormality

In Fig. 6, we summarized the results of our study and proposed the following mechanism. During the XFM culture conditions, COL coating promotes the initial attachment, stable growth, and survival of DPSCs. In a hypoxic environment (overconfluence), cell survival and sustained proliferative potential are achieved through HIF-1α-dependent production of COL1 via integrins α2β1/α11β1 interactions, allowing cells to retain stemness and chromosomal stability. Without COL coating, XFM cells display mitochondrial abnormality and undergo apoptosis.

The conventional XFM culture is a highly productive method for isolation and expansion of donor-derived DPSCs during the monolayer culture and passaging at subconfluency [37]. On the other hand, here, we show that applying COL coating method to the XFM culture ensures safer and predictable handling of DPSCs and has three main advantage points. First, the initial cell adhesion and subsequent growth are promoted after isolating and passaging the COL-XFM cells. Second, the COL-XFM cells in overconfluent/multilayered state can be protected from apoptotic death, avoiding reductions of cell yield. Third, overconfluent COL-XFM cells can be subcultured and further expanded while maintaining stemness. Therefore, the COL coating method is effective for establishing safe and reliable cell culture protocols using XFM, based on the following points: (1) it allows consistent and predictable donor-derived cell isolation and passaging in XFM culture; (2) it allows longer culture periods, resulting in overconfluence and cell yields approximately two-fold higher at a single time-point (i.e., without passaging); and (3) it allows reduction of cell-passage frequency, avoiding cell damage due to trypsin−EDTA exposure. Furthermore, this protocol avoids the exposure of DPSCs to the xenogeneic serum (i.e., FBS) throughout the whole culture period, including donor-tissue collection (transport to the laboratory), cell isolation (primary culture), expansion (subculture), and storage (cryopreservation) [37]. Hence, our current research strategy is fully committed to translational donor-derived/tissue-specific stem cell medicine based on cell isolation, expansion, and banking for future use.

This study of COL-XFM multilayered cultures, a COL1-rich stem cell niche, demonstrated the existence of the HIF-1α-dependent ECM production pathway and confirmed the role of ECM–integrin interactions in cell survival, retention of stemness, and chromosomal stability. In contrast, in the conventional XFM multilayers, the absence of COL1 resulted in cell death (anoikis), accompanied by mitochondrial abnormality. DPSCs are only one example of typical donor-derived MSC sources; therefore, these current findings are highly applicable to XFM culture of other MSCs, especially BMSCs [47, 52]. A recent study reported that collagen plays an important role in BMSCs proliferation, survival, and adhesion, compared to other coating substrates, such as fibronectin and poly-l-lysine [55]. Furthermore, other studies demonstrated that native ECM produced by the BMSCs, the matrix consisting primarily of collagen, efficiently supported stem cell properties, such as clonogenicity, cell growth, and osteogenic differentiation potential [56, 57]. Therefore, further investigation of regulatory pathways involved in DPSCs survival and stemness maintenance provides methodological and practical insights into tissue-specific stem cell biology and donor-derived stem cell medicine, contributing to the development of safe and predictable MSC-based therapies in regenerative medicine.

## Conclusion

COL coating of the cultureware enables us to achieve consistent cell isolation and robust expansion of donor-derived DPSCs, even after reaching confluence, leading to increased yield of XFM-cultured cells. In the COL-XFM cultures at overconfluency, HIF-1α-dependent expression of COL1 and integrin-mediated anchorage to COL1 are involved in cell survival and sustained proliferative potential, contributing to stem cell properties, as well as chromosomal stability after subculture. This readily and widely applicable method promises a safe and reliable XFM culture in order to establish appropriate procedures for isolating, expanding, and banking donor-derived cell source for stem cell research and therapy.

## Supplementary information

**Additional file 1: Figure S1.** Phase-contrast images of DPSCs cultured in xenogeneic serum-free culture conditions on the coated substrates as follows: type I collagen supplied by (a) Nitta Gelatin and (b) Corning; fibronectin supplied by (a) Irvine Scientific and (b) Corning; laminin supplied by (a) Corning and (b) BD Biosciences and poly-D-lysine supplied by Corning. In the initial cell growth test, cells were seeded at 5 × 10^3^ cells/cm^2^ (conventional cell density when passaging) and the images were taken on day 4 post-seeding. Scale bars, 200 μm.

**Additional file 2: Figure S2.** Phase-contrast images and quantification of DPSCs cultured on type I collagen and fibronectin in xenogeneic serum-free culture conditions. In cell adherent test, cells were seeded at 2 × 10^3^ cells/cm^2^ (a sparse cell density) and the images were taken on day 2 post-seeding. Scale bars, 200 μm. ** *p* < 0.01.

**Additional fil 3: Figure S3.** Immunocytostaining of the HIF-1α localization in COL-XFM and XFM cells cultured in the presence of YC-1 under cobalt chloride-induced hypoxia conditions. Single cell analysis. Single channels (HIF-1α, DAPI, and vimentin) are shown in gray. The fluorescence intensities of HIF-1α (green) and nucleus (DAPI, magenta) are depicted by the dashed line (yellow) in a single cell, cell morphology was determined by vimentin (red), as shown in the merged images. Scale bars, 50 μm.

**Additional file 4: Figure S4.** Presence of collagen types IV (COL4), VII (COL7), and fibronectin (FN) in COL-XFM and XFM cultures on day 20 post-seeding (overconfluent/multilayered state) was determined by immunohistofluorescence. The single channels (COL4, COL7, FN, and DAPI) are shown in gray and all nuclei in the merged channel are shown in magenta (DAPI). Scale bars, 100 μm.

**Additional file 5: Figure S5.** Cell size and cell internal complexity/granularity were determined by flow cytometry analysis using forward scatter (FSC-A) and side scatter (SSC-A) parameters, respectively, in both COL-XFM and XFM multilayers on day 20 post-seeding.

**Additional file 6: Figure S6.** Flow cytometry of the reference samples. **(A):** Cells were dissociated from the confluent COL-XFM cultures on day 10 and the suspension of live cells were gated in the forward scatter (FSC-A) and side scatter (SSC-A) dot plots to eliminate cell debris and aggregates. **(B):** For JC-1 staining, unstained cells were used as a negative control to determine background levels and autofluorescence. Cells cultured for 7 days without media change were used as a positive control: damaged mitochondria were confirmed by decrease of JC-1 fluorescence aggregates (and increase of JC-1 monomers).

## Data Availability

The datasets supporting the conclusions of this article are included within the article and its additional files.

## Abbreviations

DPSCs: Dental pulp stem cells
MSCs: Mesenchymal stem cells
FBS: Fetal bovine serum
XFM: Xenogeneic serum-free culture medium
EDTA: Ethylenediaminetetraacetic acid
TEM: Transmission electron microscope
SD: Standard deviation
PFA: Paraformaldehyde
RT: Room temperature
HE: Hematoxylin and eosin
HIF-1α: Hypoxia-inducible factor-1 alpha
Ig: Immunoglobulin
DAPI: 4′,6-Diamidino-2-phenylindole
RT-PCR: Reverse transcription-polymerase chain reaction
CoCl_2_: Cobalt chloride
COL1: Collagen type I
PB: Phosphate buffer solution
TUNEL: Terminal deoxynucleotidyl transferase-mediated deoxyuridine triphosphate nick end labeling
COL: Type I collagen
COL-XFM cells: DPSCs cultured on COL in XFM
XFM cells: DPSCs cultured on fibronectin in XFM
ECM: Extracellular matrix
