# Supplementary figures and images for: Type I collagen facilitates safe and reliable expansion of human dental pulp stem cells in xenogeneic serum-free culture
Source: Stem Cell Res Ther. 2020 Jul 14;11:267. doi: 10.1186/s13287-020-01776-7 (PMC7359624; doi:10.1186/s13287-020-01776-7)

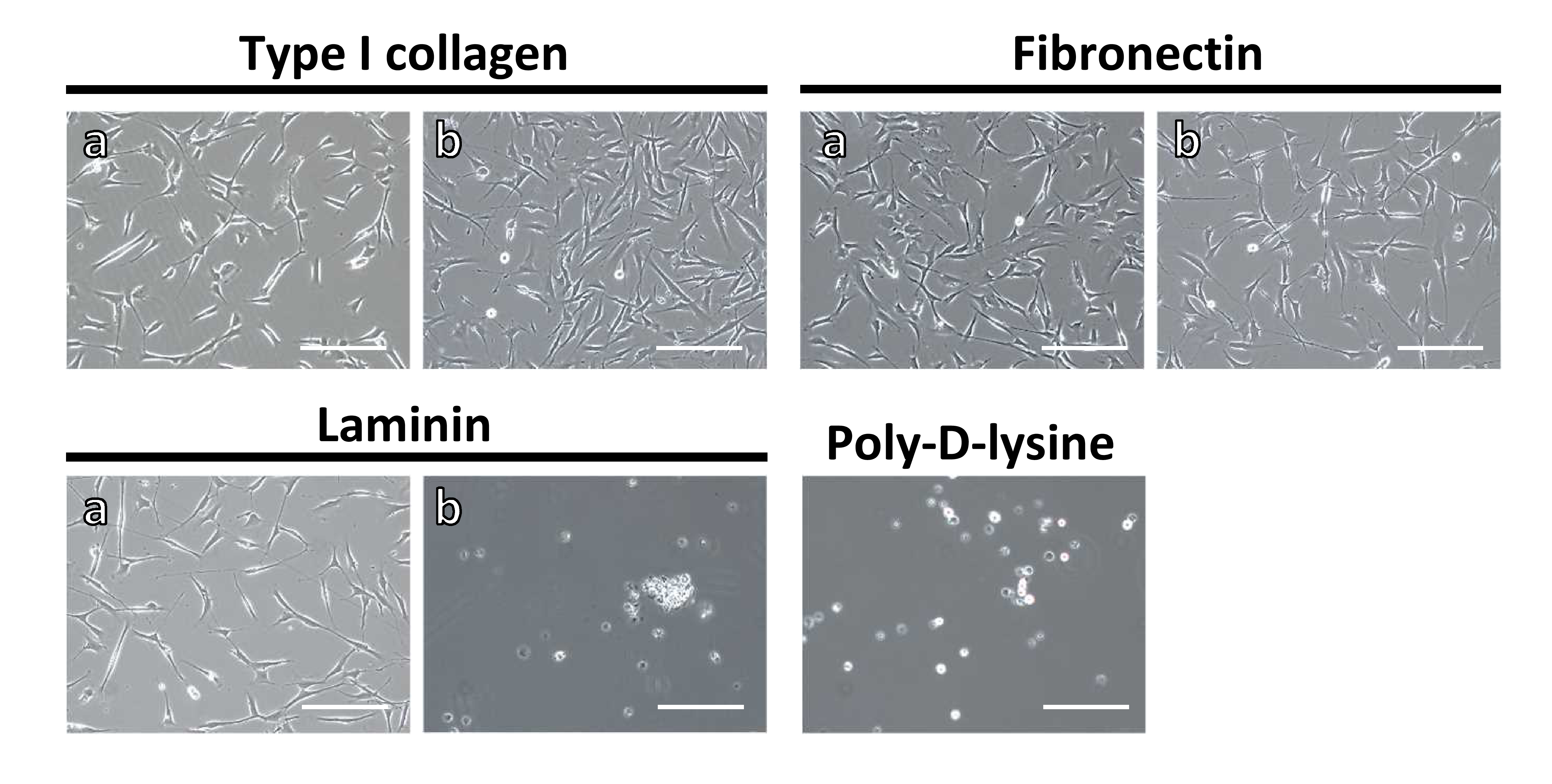

Supplement: Supplementary file 1 — Additional file 1: Figure S1. Phase-contrast images of DPSCs cultured in xenogeneic serum-free culture conditions on the coated substrates as follows: type I collagen supplied by (a) Nitta Gelatin and (b) Corning; fibronectin supplied by (a) Irvine Scientific and (b) Corning; laminin supplied by (a) Corning and (b) BD Biosciences and poly-D-lysine supplied by Corning. In the initial cell growth test, cells were seeded at 5 × 10^3^ cells/cm2 (conventional cell density when passaging) and the images were taken on day 4 post-seeding. Scale bars, 200 μm. [file 13287_2020_1776_MOESM1_ESM.tif]

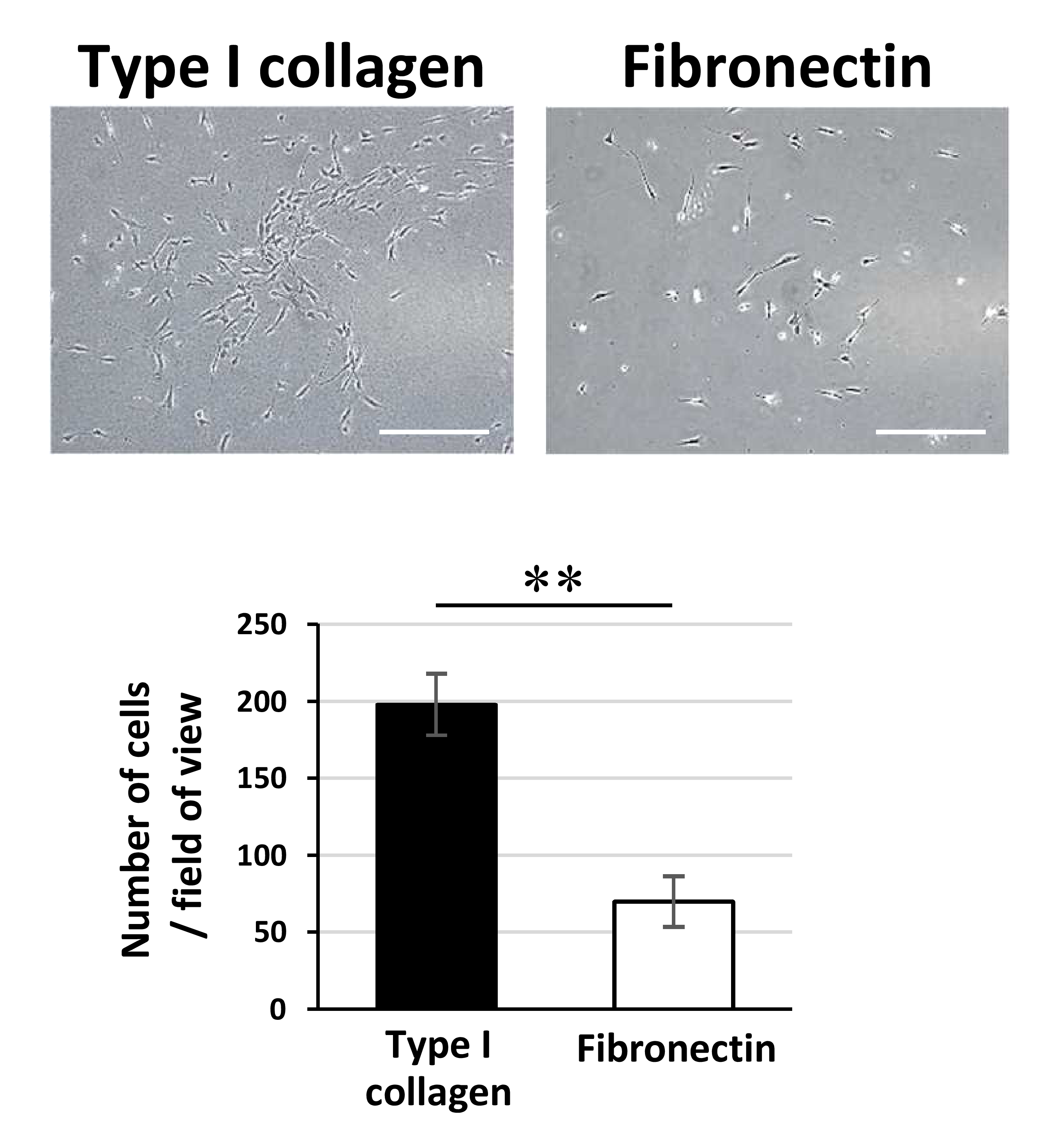

Supplement: Supplementary file 2 — Additional file 2: Figure S2. Phase-contrast images and quantification of DPSCs cultured on type I collagen and fibronectin in xenogeneic serum-free culture conditions. In cell adherent test, cells were seeded at 2 × 10^3^ cells/cm2 (a sparse cell density) and the images were taken on day 2 post-seeding. Scale bars, 200 μm. ** p < 0.01. [file 13287_2020_1776_MOESM2_ESM.tif]

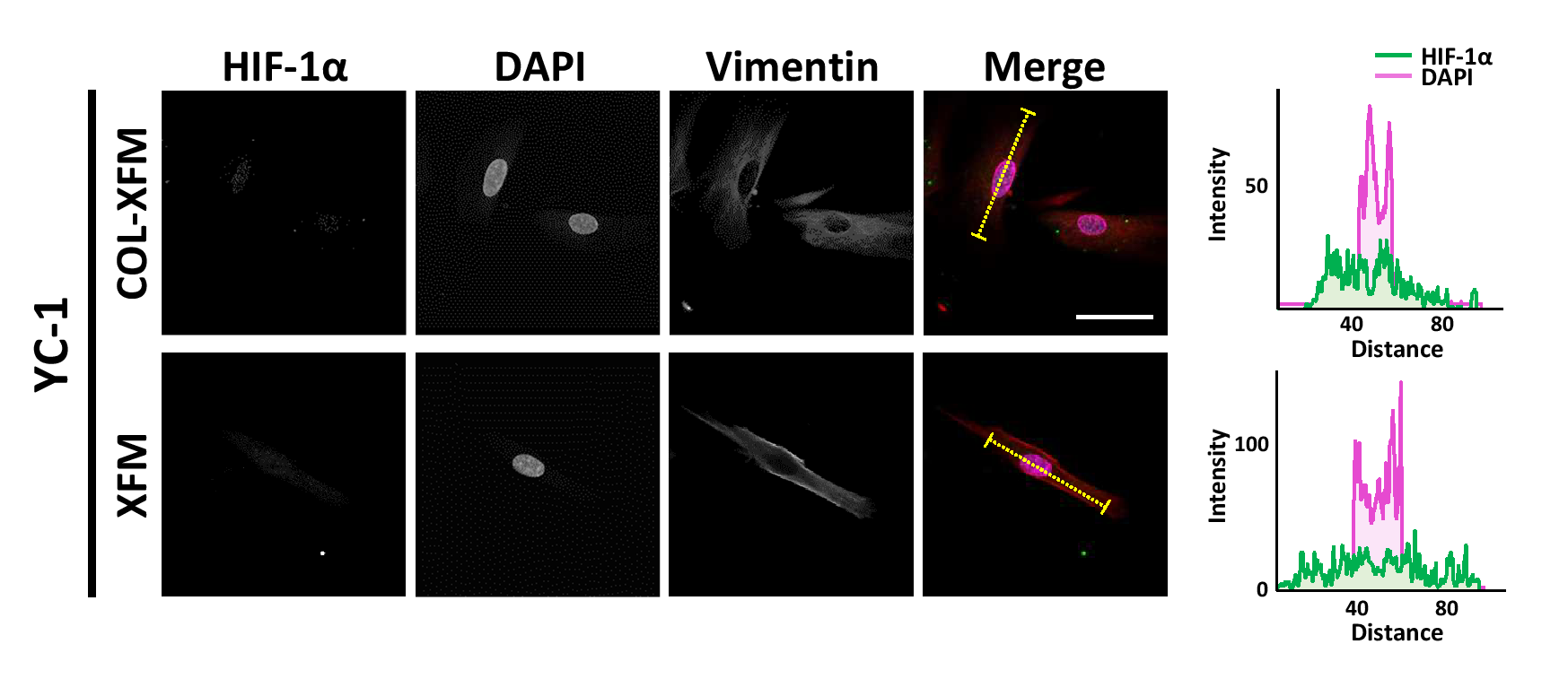

Supplement: Supplementary file 3 — Additional fil 3: Figure S3. Immunocytostaining of the HIF-1α localization in COL-XFM and XFM cells cultured in the presence of YC-1 under cobalt chloride-induced hypoxia conditions. Single cell analysis. Single channels (HIF-1α, DAPI, and vimentin) are shown in gray. The fluorescence intensities of HIF-1α (green) and nucleus (DAPI, magenta) are depicted by the dashed line (yellow) in a single cell, cell morphology was determined by vimentin (red), as shown in the merged images. Scale bars, 50 μm. [file 13287_2020_1776_MOESM3_ESM.tif]

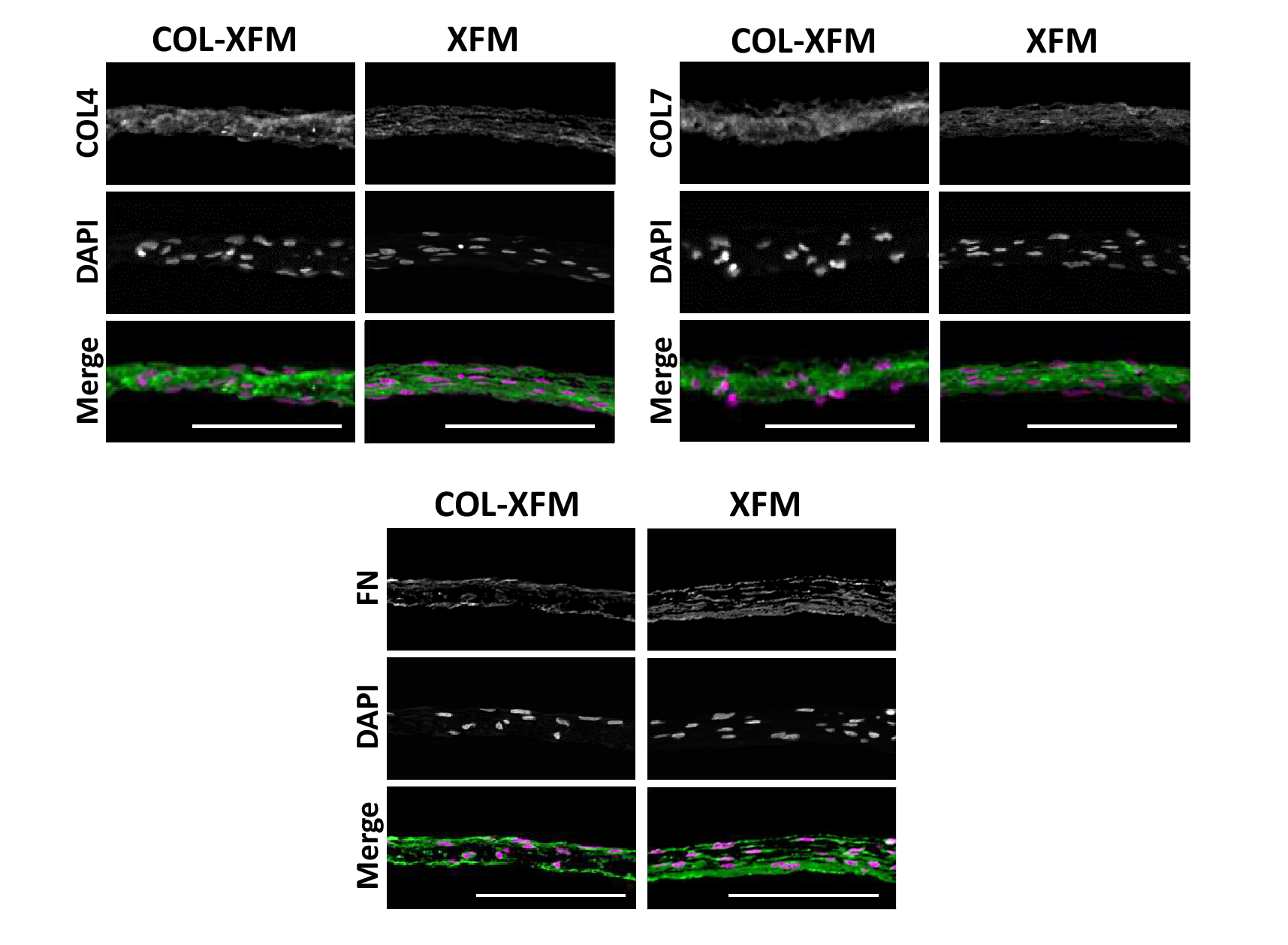

Supplement: Supplementary file 4 — Additional file 4: Figure S4. Presence of collagen types IV (COL4), VII (COL7), and fibronectin (FN) in COL-XFM and XFM cultures on day 20 post-seeding (overconfluent/multilayered state) was determined by immunohistofluorescence. The single channels (COL4, COL7, FN, and DAPI) are shown in gray and all nuclei in the merged channel are shown in magenta (DAPI). Scale bars, 100 μm. [file 13287_2020_1776_MOESM4_ESM.tif]

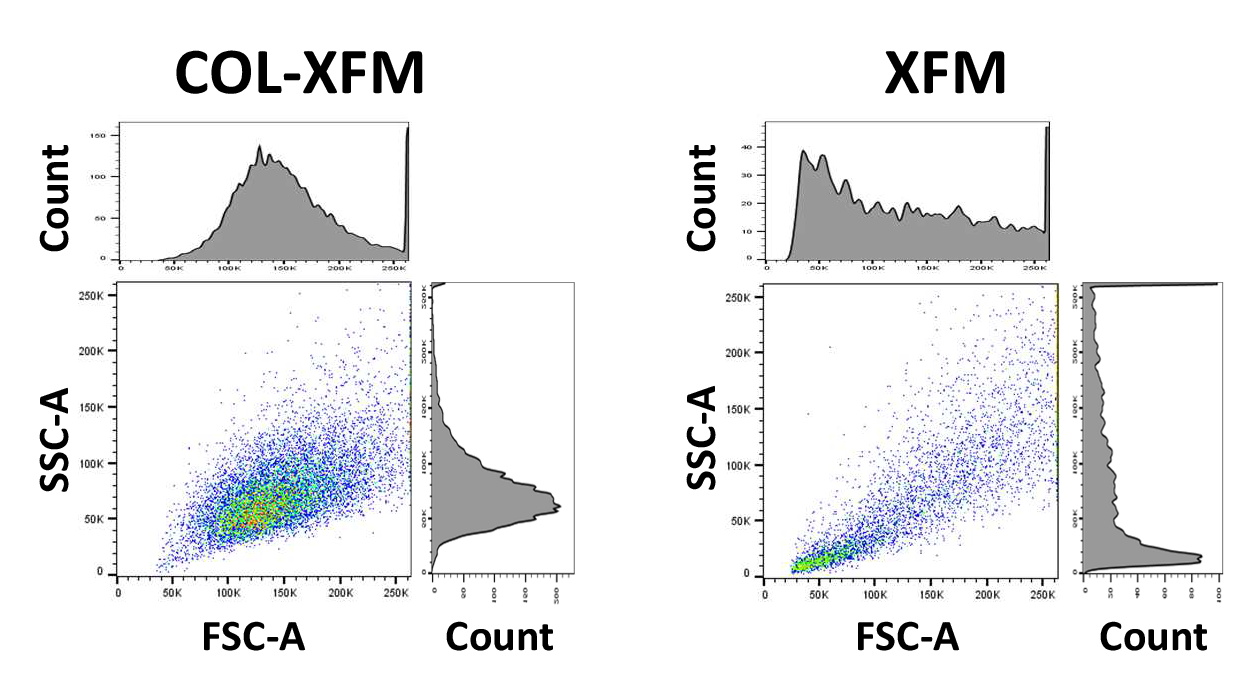

Supplement: Supplementary file 5 — Additional file 5: Figure S5. Cell size and cell internal complexity/granularity were determined by flow cytometry analysis using forward scatter (FSC-A) and side scatter (SSC-A) parameters, respectively, in both COL-XFM and XFM multilayers on day 20 post-seeding. [file 13287_2020_1776_MOESM5_ESM.tif]

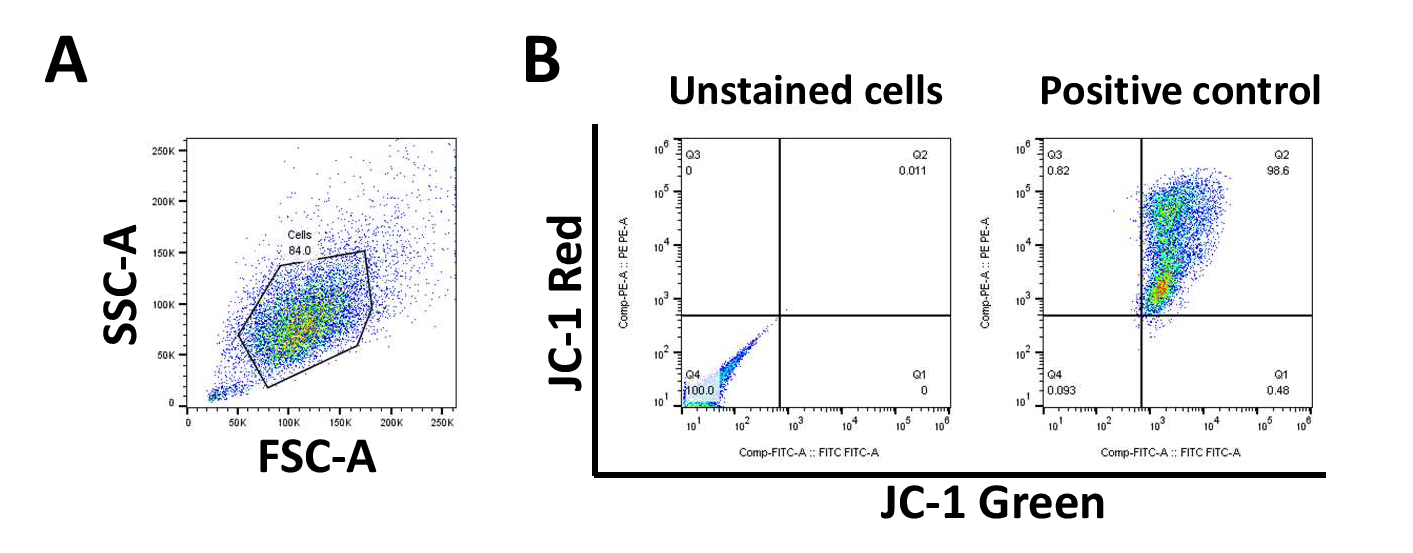

Supplement: Supplementary file 6 — Additional file 6: Figure S6. Flow cytometry of the reference samples. (A): Cells were dissociated from the confluent COL-XFM cultures on day 10 and the suspension of live cells were gated in the forward scatter (FSC-A) and side scatter (SSC-A) dot plots to eliminate cell debris and aggregates. (B): For JC-1 staining, unstained cells were used as a negative control to determine background levels and autofluorescence. Cells cultured for 7 days without media change were used as a positive control: damaged mitochondria were confirmed by decrease of JC-1 fluorescence aggregates (and increase of JC-1 monomers). [file 13287_2020_1776_MOESM6_ESM.tif]
